# Genetic Architecture of Parkinson's Disease in the Indian Population: Harnessing Genetic Diversity to Address Critical Gaps in Parkinson's Disease Research

**DOI:** 10.3389/fneur.2020.00524

**Published:** 2020-06-18

**Authors:** Roopa Rajan, K. P. Divya, Rukmini Mridula Kandadai, Ravi Yadav, Venkata P. Satagopam, U. K. Madhusoodanan, Pankaj Agarwal, Niraj Kumar, Teresa Ferreira, Hrishikesh Kumar, A. V. Sreeram Prasad, Kuldeep Shetty, Sahil Mehta, Soaham Desai, Suresh Kumar, L. K. Prashanth, Mohit Bhatt, Pettarusp Wadia, Sudha Ramalingam, G. M. Wali, Sanjay Pandey, Felix Bartusch, Maximilian Hannussek, Jens Krüger, Ashwin Kumar-Sreelatha, Sandeep Grover, Peter Lichtner, Marc Sturm, Jochen Roeper, Volker Busskamp, Giriraj R. Chandak, Jens Schwamborn, Pankaj Seth, Thomas Gasser, Olaf Riess, Vinay Goyal, Pramod Kumar Pal, Rupam Borgohain, Rejko Krüger, Asha Kishore, Manu Sharma, The Lux-GIANT Consortium

**Affiliations:** ^1^Department of Neurology, All India Institute of Medical Sciences, New Delhi, India; ^2^Sree Chitra Tirunal Institute for Medical Sciences, Trivandrum, India; ^3^Department of Neurology, Nizam's Institute of Medical Sciences, Hyderabad, India; ^4^National Institute of Mental Health and Neurosciences (NIMHANS), Bengaluru, India; ^5^Luxembourg Centre for Systems Biomedicine, University of Luxembourg, Luxembourg, Luxembourg; ^6^ELIXIR-Luxembourg Node, Belvaux, Luxembourg; ^7^Movement Disorders Clinic, Global Hospitals, Mumbai, India; ^8^All India Institute of Medical Sciences, Rishikesh, India; ^9^Goa Medical College, Panaji, India; ^10^Institute of Neurosciences, Kolkata, India; ^11^Lourdes Hospital, Kochi, India; ^12^Narayana Hrudayalaya Multispeciality Hospital, Bangalore, India; ^13^Department of Neurology, PGIMER, Chandigarh, India; ^14^Shree Krishna Hospital and Pramukhswami Medical College, Karamsad, India; ^15^Department of Neurology, Vijaya Health Centre, Chennai, India; ^16^Center for Parkinson's Disease and Movement Disorders, Vikram Hospital, Bangalore, India; ^17^Kokilaben Dhirubhai Ambani Hospital, Mumbai, India; ^18^Jaslok Hospital, Mumbai, India; ^19^Department of Community Medicine, PSG Institute of Medical Sciences and Research, Coimbatore, India; ^20^Neurospecialities Centre, Belgaum, India; ^21^Department of Neurology, G. B. Pant Institute of Medical Education and Research, New Delhi, India; ^22^Zentrum für Datenverarbeitung (ZDV), University of Tubingen, Tübingen, Germany; ^23^Centre for Genetic Epidemiology, Institute for Clinical Epidemiology and Applied Biometry, University of Tubingen, Tübingen, Germany; ^24^Helmholtz Zentrum München, German Research Center for Environmental Health, Institute of Human Genetics, Neuherberg, Germany; ^25^Institute for Medical Genetics and Applied Genomics, University of Tubingen, Tübingen, Germany; ^26^Institute of Neurophysiology, Goethe University Frankfurt, Frankfurt, Germany; ^27^Department of Ophthalmology, Universitäts-Augenklinik Bonn, University of Bonn, Bonn, Germany; ^28^Centre for Cellular and Molecular Biology, Hyderabad, India; ^29^National Brain Research Centre, Gurugram, India; ^30^Department of Neurodegenerative Diseases, Hertie Institute for Clinical Brain Research, University of Tübingen, Tübingen, Germany; ^31^Medanta the Medicity, Gurgaon, India; ^32^Transversal Translational Medicine, Luxembourg Institute of Health (LIH), Strassen, Luxembourg

**Keywords:** Parkinson's disease, genetic diversity, genome-wide association study, common genetic variation, biobank

## Abstract

Over the past two decades, our understanding of Parkinson's disease (PD) has been gleaned from the discoveries made in familial and/or sporadic forms of PD in the Caucasian population. The transferability and the clinical utility of genetic discoveries to other ethnically diverse populations are unknown. The Indian population has been under-represented in PD research. The Genetic Architecture of PD in India (GAP-India) project aims to develop one of the largest clinical/genomic bio-bank for PD in India. Specifically, GAP-India project aims to: (1) develop a pan-Indian deeply phenotyped clinical repository of Indian PD patients; (2) perform whole-genome sequencing in 500 PD samples to catalog Indian genetic variability and to develop an Indian PD map for the scientific community; (3) perform a genome-wide association study to identify novel loci for PD and (4) develop a user-friendly web-portal to disseminate results for the scientific community. Our “hub-spoke” model follows an integrative approach to develop a pan-Indian outreach to develop a comprehensive cohort for PD research in India. The alignment of standard operating procedures for recruiting patients and collecting biospecimens with international standards ensures harmonization of data/bio-specimen collection at the beginning and also ensures stringent quality control parameters for sample processing. Data sharing and protection policies follow the guidelines established by local and national authorities.We are currently in the recruitment phase targeting recruitment of 10,200 PD patients and 10,200 healthy volunteers by the end of 2020. GAP-India project after its completion will fill a critical gap that exists in PD research and will contribute a comprehensive genetic catalog of the Indian PD population to identify novel targets for PD.

## Background

Parkinson's disease (PD) is the second most common neurodegenerative disorder in adults over the age of 60 years (1). According to the Global Burden of Disease study (2018), the worldwide burden of PD has more than doubled over the past two decades from 2.5 million patients in 1990–6.1 million patients in 2016 (2). India is home to nearly 0.58 million persons living with PD as estimated in 2016, with an expected major increase in prevalence in the coming years (2). Despite the large number of people affected with PD, insights into the underlying genetic and environmental risk factors specific to the Indian population are limited. This is in contrast to the Caucasian population in whom easy access to the patient cohort and the population homogeneity have driven initial large scale genome-wide studies (3, 4). Despite the success, the constraints of performing studies in a single homogenous population became apparent as well. This is because the Caucasian population contains only a subset of genetic diversity (5). Populations vary in terms of allele frequency, linkage disequilibrium (LD) patterns, and differences in effect estimates. This provides a scientific rationale that no single population is sufficient to fully uncover the variants underlying disease in all populations, and makes it imperative to pursue genetic research in diverse populations to capture the genetic diversity of a disease.

About 5–10% of PD is monogenic and inherited in an autosomal dominant or recessive manner. The large majority of patients have a sporadic disease. To date, 90 PD loci have been identified explaining a missing heritability in a range of 16–36% (3). It is also increasingly recognized that additional loci with varying degrees of minor allele frequency and effect size remain to be discovered which might account for the remaining missing heritability. Most of the PD loci have been identified in cohorts that are heavily biased toward persons with Caucasian ancestry (3, 4). This generates issues of reproducibility in a global context. For instance, variants in leucine-rich repeat kinase 2 (*LRRK2*), glucocerebrosidase (*GBA*), and alpha-synuclein (*SNCA*) genes identified in the western population have been shown to pose negligible risk to the Indian patients (6–9). Novel variants in the known genes or novel genes may be associated with PD risk in the genetically more diverse Indian population (10). Variations in allele frequency in genetically heterogeneous populations may provide adequate power to GWA studies with smaller sample sizes for the enriched loci. For example, the discovery of an association at a new putative locus at chr1 (PARK16) in the Japanese population for PD underscores the need to study ethnically diverse populations. The associated SNP, rs823128, which was shown to be protective against the development of PD specifically in the Asian population has a minor allele frequency ~20% in the Japanese population as compared to only 3% in the Caucasian population (11). With this minor allele frequency, individual GWAS in the Caucasian populations had very little power to detect an association, even though the SNP was well-tagged with arrays. The 1,000 Genomes project which uses the combinatorial approach of exome and whole-genome sequencing suggests that individuals from different populations carry different profiles of rare and common variants and that low-frequency variants show substantial geographic differentiation, thus arguing in favor of diversifying genetic research especially in populations which have so far been underrepresented in gene mapping such as the Indian population (12). In addition to the potential for new gene discovery, the inclusion of ethnically diverse cohorts provides an opportunity to cross-validate newly identified loci, which has direct implications for the global applicability and scalability of potential novel therapeutic targets.

We initiated the Genetic Architecture of Parkinson disease in India (GAP-India) project to provide for the first time, a large-scale genetic catalog of the Indian PD population. This paper describes the design of the GAP-India project including the study sites, subject recruitment, clinical assessments, biospecimens processing, plan for data analysis and sharing, capacity building, and the ethical and regulatory frameworks within which we operate.

## Study Design

**Figure 1:**
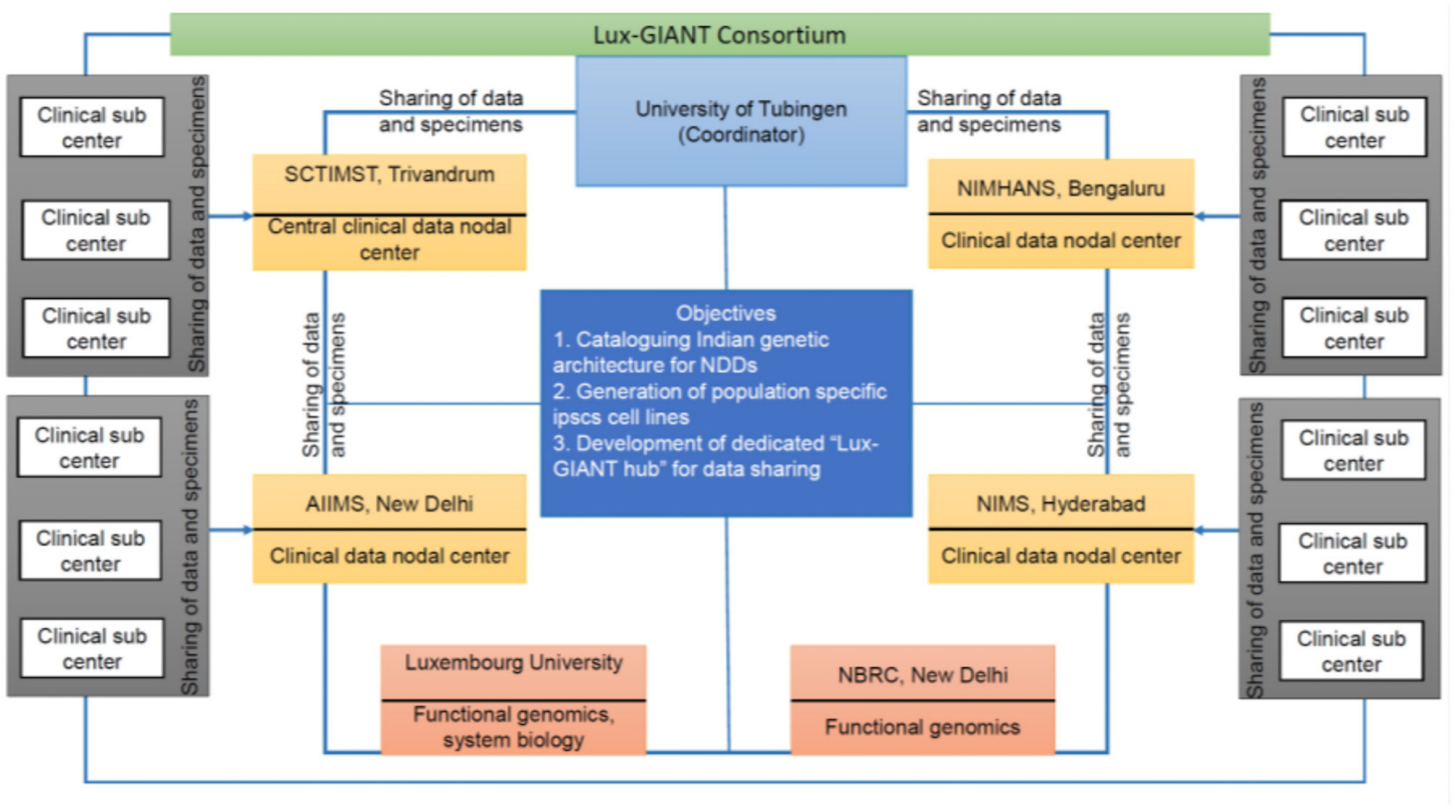
Overall flow chart describing the details of the Lux-GIANT consortium. The consortium follows the “hub-spoke” model. The Lux-GIANT has established three main cores: genomics, clinical, and functional genomics. The University of Tubingen is the coordinator site. Luxembourg site aims to strengthen functional genomics and system biology. Functional core from India, National Brain Research Center, will be responsible for developing and maintaining iPSCs. Four main clinical-nodes capturing most of India are formed. These four nodes are connected to clinical sub-centers which span throughout India. The “central clinical node” aims to streamline the administrative process, which is required for clearance and sample shipment. The different nodes within India are connected with the main “central clinical node” for continuous update of cohort and members.

**Figure 2:**
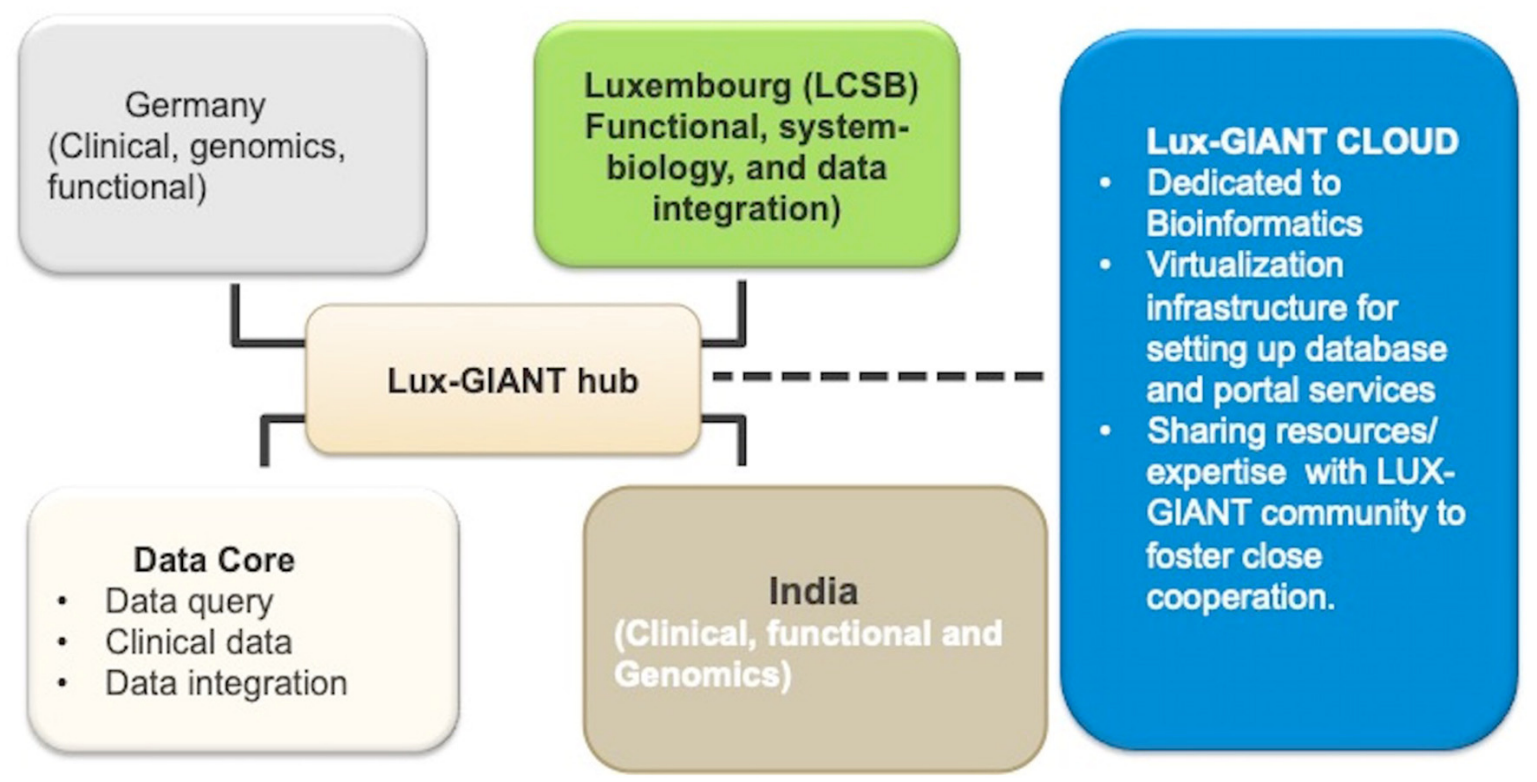
Clinical, genomics, bioinformatics, and functional cores in the Lux-GIANT consortium.

GAP-Indiastudy aims to understand the genetic architecture of PD in the Indian population through large scale sample collection and federated data analysis models. The study aims to collect pan-Indian genetic and phenotypic data and will develop one of the largest clinico-genomic PD resources for the scientific community from India. To achieve our objectives, we have formed a trilateral consortium, the Luxembourg-German-Indian Alliance on Neurodegenerative diseases and Therapeutics (Lux-GIANT) (Figure 1). The “knowledge-sharing” model aims to build capacity and exchange programs to integrate clinical/genetic centers and harmonize data collection with Luxembourg and German centers. Lux-GIANT follows a decentralized model and based on expertise, different cores have been created (Figure 2). For example, the central clinical core in India is established at the Sree Chitra Tirunal Institute for Medical Sciences and Technology (SCTIMST), Trivandrum, Kerala. Given the diversity and vastness of India, apart from the central clinical core, three high volume academic movement disorders centers across India have been identified and established as nodal centers to recruit participants with a pan-India representation. These four clinical nodes are further connected to twenty clinical sub-centers which are spread throughout India. The central clinical core is responsible for coordinating patient recruitment and biospecimen collection with nodal centers. The nodal centers supervise the patient recruitment and biospecimen collection at the sub-centers. Similarly, genomics, functional and bioinformatics cores have been established in Luxembourg, Germany, and India. The functional core at the National Brain Research Center, Manesar, India, is mandated to develop the Lux-GIANT iPSCs biobank. The functional core in India coordinates its activities with the functional cores in Germany and Luxembourg. This has been done to share protocols and develop common functional protocols to perform functional studies. The array processing will be done at the Center for Cellular and Molecular Biology (CCMB), Hyderabad. The central genomics/bioinformatics core at Tubingen will coordinate data generation with the local center and subset of samples will be processed in Germany for quality control purposes.

## Projected Cohort and Study Sites

The GAP-India study revolves around a network of clinical sites in India organized in a “hub and spoke” manner. Patient recruitment at each nodal or sub-center will be supervised by a neurologist with specific expertise in Movement Disorders. Subjects will be enrolled at all the sub-centers and the four nodal centers (SCTIMST, Trivandrum; All India Institute of Medical Sciences, New Delhi; National Institute for Mental Health and Neurosciences, Bengaluru and Nizams Institute of Medical Sciences, Hyderabad). The nodal centers are all high-volume academic centers with established movement disorders programs. The sub-centers include additional public sector teaching hospitals, larger multispecialty hospitals, and neurology clinics in the private sector.

Genetic evidence indicates that most Indians descended from a mixture of two divergent populations: Ancestral North Indians (related to Central Asians, Middle Easterners, and Europeans) and Ancestral South Indians (not closely related to other genetic groups) and almost all the current inhabitants are admixtures of these two broad groups to varying extents (13). Within the population, allele frequency changes between subgroups are larger than in European populations, owing to founder effects maintained by a transition to endogamy about 1,900–4,200 years ago (14). The 1,000 genomes project contains about 500 genomes from the Indian subcontinent (including India and geographically neighboring countries), from five diverse linguistic groups, yet the Ancestral North Indian component is prominent in this dataset (15). Within the linguistic groups too, population substructures were evident suggesting that careful matching of cases and controls from within the same ethno-linguistic groups is necessary to avoid false positive associations.

Geographical locations of the enrolling sites in India were chosen to consider this unique population structure and to enable a pan-Indian representation (Supplementary Figure 1). The study aims to enroll 10,200 PD patients and 10,200 healthy volunteers over 1 year. Furthermore, GAP-India aims to develop a cohort of 25,000 cases and 25,000 controls by 2024. The four nodal centers will directly enroll about 6,000 patients and the remaining subjects will be enrolled at the sub-centers. The sample size was chosen to take into consideration the statistical power to detect a risk associated variant in GWAS as well as the feasibility of attaining it within the timeframe of the project. The extensive multi-centric nature of the project helps in covering diverse genetic subgroups and meeting recruitment goals within the timelines.

## Subject Recruitment

Subjects will be recruited from the Movement Disorder clinics or Neurology clinics run by the PIs of nodal and sub-centers. A detailed history and systemic and general neurological examination will be performed in all subjects. Research staff at all recruiting centers will be trained in the standard operating procedures including clinical assessments and familiarized with online data entry systems before site initiation. Subjects who meet all the following inclusion criteria will be recruited in the patient group: (1) clinical diagnosis of PD as per United Kingdom Parkinson's Disease Society Brain Bank (UKPDSBB) diagnostic criteria (16), (2) age more than 18 years and (3) Asian Indian ethnicity. Subjects meeting any of the following criteria will be excluded: (1) cognitive or psychiatric dysfunction sufficiently severe enough to impair the patient's ability to provide informed written consent (2) red flags or additional neurological signs raising suspicion of atypical Parkinsonism. Patients who have previously undergone surgical procedures such as pallidotomy, thalamotomy, or Deep Brain Stimulation will not be excluded. Healthy volunteers will be recruited through advertisements displayed on the hospital campus. They will be gender-matched and should belong to the same geographic–ethnic background as the patients. A detailed history and standard neurological examination will be performed before inclusion as controls. Volunteers with a family history of PD or other neurodegenerative diseases will be excluded from the control group. All subjects will be recruited after obtaining written informed consent and with the approval of the Institutional Ethics Committee. Centralized monitoring of recruitment rates and fidelity to operating procedures will be done by the clinical nodal center, SCTIMST.

## Clinical Assessments and Biospecimen Collection

Trained personnel at each clinical site will collect clinical and demographic data. The demographic data collected includes information on the geographical origin within India. Structured questions will capture information related to environmental exposures known to be associated with PD including pesticides, fungicides, insecticides, and other chemicals, smoking, caffeine, and head injury. Patients will be asked to report if they ever held a job requiring exposure to pesticides, herbicides, fungicides, insecticides, rodenticides, and fumigants and whether they were exposed to these chemicals at their place of residence through self-use or via another person. Lifetime smoking history of 100 or more cigarettes will be documented. History of head injury or concussion including falls, sporting activities, violence, and car or other accidents in childhood or adulthood will be queried. Patients will also be asked about exposure to caffeinated coffee in quantities more than once per week for 6 months or longer. Years of education will be documented. Furthermore, our environmental exposure data collection will align with the environmental questionnaire from the Genetic-Epidemiology of Parkinson disease (GEoPD) consortium to harmonize the dataset across the ongoing studies. A structured history and clinical examination will be conducted to collect data regarding onset symptoms, motor fluctuations, and dyskinesias, medications, and non-motor symptoms. Non-motor symptoms included in the interview are cognitive impairment, psychosis, depression, sweating abnormalities, seborrhea, sleep disorders including REM behavioral disorder, restless legs, hyposmia, orthostatic hypotension, constipation, dysphagia, and urinary/fecal incontinence. Data from Computerized Tomography (CT), Magnetic Resonance Imaging (MRI), Dopamine Active Transporter- Single Photon Emission Computed Tomography (DaT SPECT) will be collected if available. The family history will be probed for consanguinity and to identify any known relations with PD, dementia, tremor, or other neurological disorders. For patients who have undergone functional neurosurgery, the target, time since surgery and other details will be collected. The motor symptoms at the time of recruitment will be assessed by the Unified Parkinson's Disease Rating Scale (UPDRS Parts I- IV) (17). Subjects will be screened for cognitive dysfunction using the Montreal Cognitive Assessment (MoCA) and for depression using the Beck's Depression Inventory (BDI- II) (18, 19). Validated regional language versions of MoCA will be used for non-English speaking subjects. Demographic and risk factor information will be collected from the control group. Clinical terminologies have been standardized to enable data harmonization with existing research groups and also build a phenotypic information resource for this particular population of PD patients.

The whole blood samples will be collected at each recruiting center 10–15 ml of blood samples collected in EDTA tubes will be processed for DNA extraction using the *salting-out* method. The quantity and quality of DNA will be analyzed using a micro-volume UV/visible spectrophotometer (Nanodrop, Thermo Fischer). Additionally, the quality will also be checked by agarose gel electrophoresis. Those samples with an A_260/280_ ratio of 1.8–2.0 and A_260/230_ ratio of >2.0 will be stored in 1.2 ml screwcap cryovials barcoded with a unique sample ID at SCTIMST Biobank (−80°C), in aliquots. The samples received from the nodal centers and sub-centers will be again checked (using Nanodrop and agarose gel) at SCTIMST to ensure the quality and quantity of DNA that is required for the genotyping. For sequencing, 50 μl of 50–100 ng/μl DNA will be transported in 96-well microtiter plates sealed with peelable heat seals in a waterproof container and dry ice. At the time of collection, specimens will be de-identified by avoiding any personal identifiers on the label. Specimen labels and data collection instruments will be labeled by center-specific serial numbering. No direct personal identifiers will be stored in the online data capture system and quasi-identifiers like date of birth are flagged as such and de-identified by the system before export. Only the site PIs hold identifying information if required for re-identification at a later stage. The central clinical node and other investigators with access to the online database will not have access to direct personal identifiers. Biospecimens collected at the sub-centers will be transported to the nodal centers for DNA isolation. All biospecimens are finally routed to the clinical core at SCTIMST, Trivandrum for storage and in consenting subjects, longer-term bio-banking. In keeping with the existing regulatory framework in India, to promote capacity development, array processing and genotyping will be done at the Center for Cellular and Molecular Biology, Hyderabad. The genetic core at Tübingen will perform the bioinformatics analysis and a subset of specimens will be processed at the Lux-GIANT genotyping core facility in Munich for quality control purposes. All clinical and genetic data will be stored on a shared electronic platform with access restrictions and security protocols in place. Functional validation of putative pathogenic variants including patient-specific induced pluripotent stem cell modeling will be done at the National Brain Research Center, Gurgaon, India. In this way, the study is designed to comprehensively capture clinical and genetic information from a large Indian cohort in a manner that enables integration with existing international cohorts. DNA isolation from whole blood will be done at the four nodal centers and centralized quality control monitoring at SCTIMST, Trivandrum. DNA specimens from consenting subjects will be maintained in a biorepository at SCTIMST for potential future research.

## Data Analysis

For genetic analysis, a two-stage design will be followed. Currently, the arrays available for genotyping lack in-depth genetic variability information from the Indian population. GAP-India aims to address this issue by performing whole-genome sequencing (WGS) of around 500 subjects covering the north, south, east, and west of India.

### Whole-Genome Sequencing

The data generated from whole-genome sequencing will be analyzed using the megSAP pipeline (https://github.com/imgag/megSAP) developed at the Institute of Medical Genetics and Applied Genomics, University Hospital of Tübingen (Tübingen, Germany). In brief, SeqPurge (v. 2020_03) will be used for adapter and quality trimming (20), Burrow-Wheeler Aligner mem (BWA mem (v.0.7.17) for read mapping (21), samblaster (v. 0.1.24) for duplicate removal (22), ABRA2 (v. 2.22) for indel-realignment (23), freebayes (v. 1.2.0) for calling of small variants (24), ClinCNV (v. 1.16.1) for CNV calling (25), Manta (v. 1.6.0) for structural variant calling (26), and Ensembl VEP (v. 96.3) for variant annotation (27). Furthermore, additional tools available from the ngs-bits toolset (https://github.com/imgag/ngs-bits) will be used for data cleaning.

### GWAS Analysis

#### Study Population and Genotyping

The analysis cohort will represent ~10,200 PD cases and 10,200 controls of Indian ancestry, genotyped with Illumina's Global Diversity Array (GDA) containing neurodegenerative specific content.

#### Quality Control

GenomeStudio will be used to cluster the genotyping array using the GenCall algorithm and preliminary QC will be implemented in GenomeStudio as described elsewhere (28). Data will be exported in the standard PLINK format and downstream QC procedures and statistical analysis will be conducted using the latest PLINK (http://pngu.mgh.harvard.edu/_purcell/plink) and R software packages (http://www.r-project.org/), installed on a Linux based computation resource (29). The post-GenomeStudio QC will be broadly divided into three main steps comprising of (i) Sample and genetic marker quality (ii) Population structure (iii) Genotyping consistency. Furthermore, QC will be implemented independently in each Indian subpopulation covering north, south, east, and west of India.

### Sample and Genetic Marker Quality

Firstly, all samples and SNPs with missing rate>1% will be excluded. Concerning genetic marker quality, we would exclude SNPs with MAF <0.01 and HWE *p*-value <1 × 10^−10^ in cases as well as HWE <1 × 10^−6^ in controls (30). Allele frequencies will be checked with Indian sub-populations represented in the Haplotype Reference Consortium (HRC). Furthermore, allele frequency consistency across different batches of genotyping datasets will be checked to rule out the batch effect.

### Population Structure

**Figure 3:**
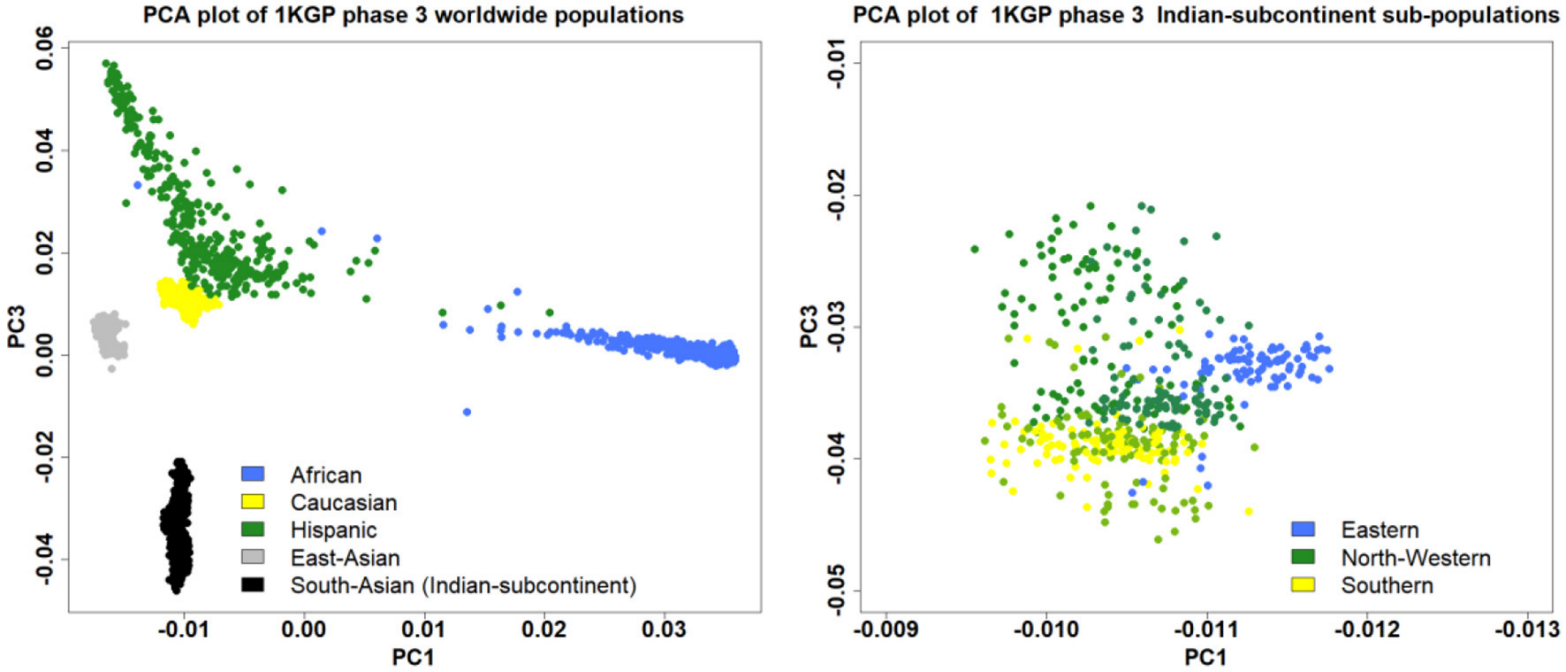
The PCA plot using the phase 3 1,000 Genomes Project showing the distribution of the South Asian population among the worldwide populations (left), and sub-populations from different regions of the Indian subcontinent (right).

Individuals deviating ±3 SD from the samples' heterozygosity rate mean will be excluded. Only those males will be included which have an X chromosome homozygosity estimate of more than 0.8. On the other hand, only those females will be included which have an X chromosome homozygosity estimate of less than 0.2. Related samples will be filtered based on identity by descent (IBD) coefficient>0.1 (31). Principal component analysis (PCA) will be used to detect population outliers using the first ten principal components and the outlier samples will be removed. We identified five populations representing the Indian subcontinent in phase 3 1,000 Genomes Project (KGP): two from the northwestern region [Gujarati Indian in Houston, TX (GIH) and Punjabi in Lahore, Pakistan (PJL)], two from Southern region [Indian Telugu in the UK (ITU) and Sri Lankan Tamil in the UK (STU)] and one from Eastern region [Bengali in Bangladesh (BEB)]. The five Indian subcontinent populations marked as South Asain population in the PCA plot of the worldwide population showed a clear demarcation emphasizing the need to diversify the genomic research in under-represented populations to identify population-specific novel genetic loci for complex diseases (Figure 3).

#### Imputation

Imputation will be carried out using the Haplotype reference consortium (HRC) as a reference panel consisting of individuals from more than 26 worldwide populations (32). The SNPs with imputation info score of less than 0.7 will be discarded.

#### Association Analysis

Post-QC and imputation, association analysis will be conducted for each sub-population using binary logistic regression analysis assuming an additive genetic model adjusting for age, sex, and relevant principal components. A conventional genome-wide significance threshold of 5 × 10–8 will be used to identify the significant SNPs. The fixed meta-analyses inverse-variance weighting of log-ORs will be implemented in METAL to combine summary statistics across all the Indian sub-populations (33). Genome inflation factor λ will be computed using the median χ^2^-statistics. Lastly, Manhattan and QQ plots will be constructed to visualize the results. All the summary statistics will be made available publicly. Heterogeneity in allelic effect sizes between different Indian sub-populations contributing to the meta-analysis will be assessed using Cochran's *Q* statistic.

#### Polygenic Prediction

We will further use genome-wide complex trait analysis (GCTA) to perform conditional and joint analysis to identify the top variants that account for heritable variation among different loci (34). Polygenic risk score profiling will be done in a standard weighted allele dose manner (35).

#### Biological Annotation

We will further integrate our GWAS summary statistics with expression and network data using Functional Mapping and Annotation of Genome-Wide Association Studies (FUMA) to perform the tissue specificity and pathway enrichment analysis (36).

The genomics/bioinformatics core in Tubingen will be responsible for data integration and analysis.

#### Data Management and Sharing

**Figure 4:**
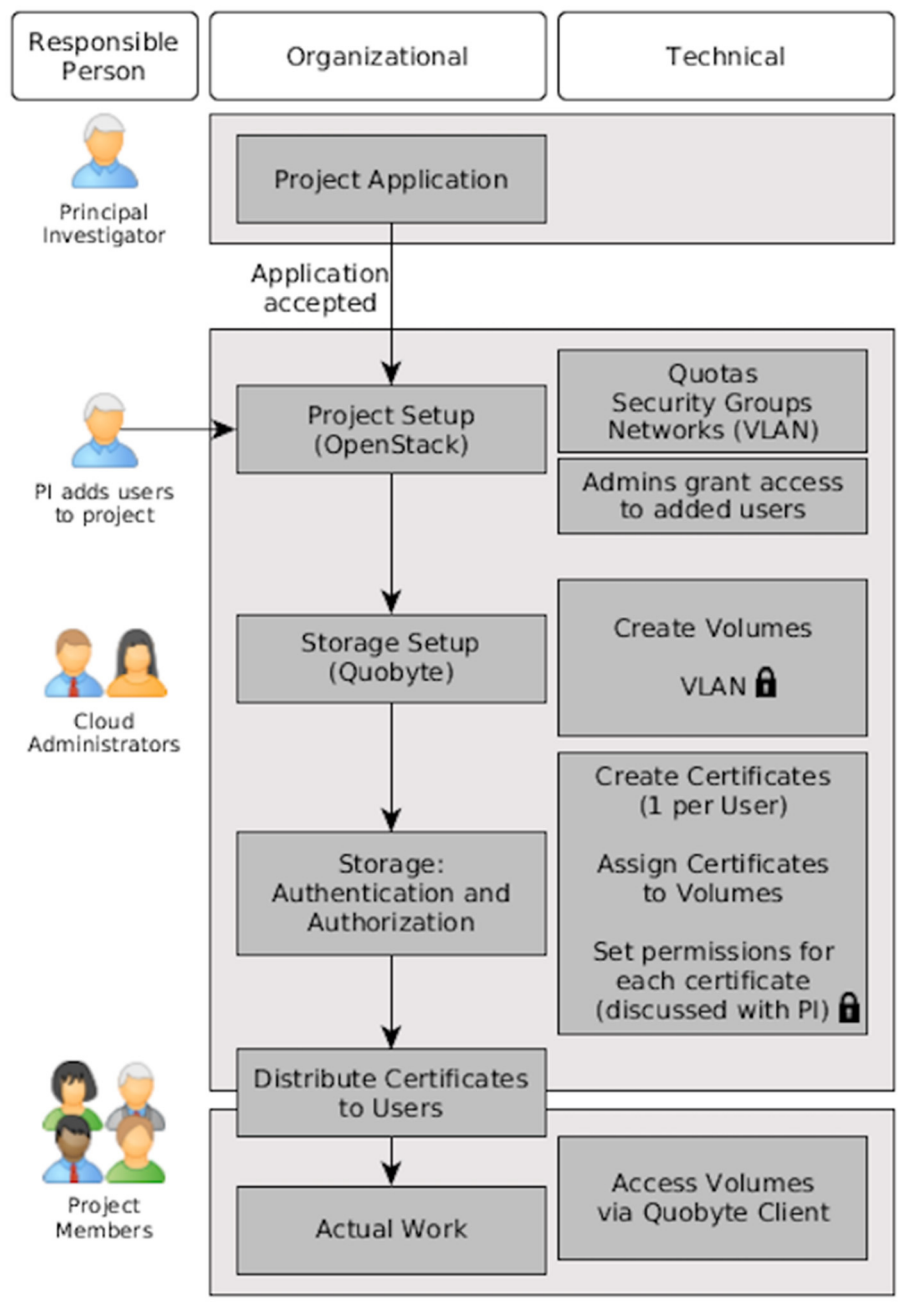
Data management and access workflows in the Lux-GIANT network.

To ensure seamless data exchange among Lux-GIANT partners, we have established a secure data management and analysis platform. At this moment this platform is equipped with REDCap, a state-of-the-art EDC (Electronic Data Capturing) system (https://www.project-redcap.org) widely used in various clinical and translational projects and is hosted at the “clinical core” site, SCTIMST. The Lux-GIANT REDCap instance is aligned with the Genetic Epidemiology of Parkinson disease (GEoPD) consortium minimal dataset Case Report Forms (CRFs). This will ensure uniform clinical data collection across various participating countries in GEoPD that are spread across five continents. This will facilitate cross-study data pooling and analysis for future multinational projects. All Indian nodal centers and sub-centers taking part in this study are collecting pseudonymized data into this secure and access-controlled instance centrally. All the identifiable information of each study participant stored separately at each site in a corresponding hospital system and only authorized clinical people from that site to have access to it. This setup is aligned with European general data protection regulations (GDPR) as well as Personal Data Protection Bill (PDP) 2019. This pseudonymized clinical data, as well as the corresponding molecular data, will be served to all Lux-GAINT partners via the “data core” site established at Tubingen by leveraging the infrastructure established by the German Network for Bioinformatics and Infrastructure (de.NBI, https://cloud.denbi.de). Through the dashboard of the de.NBI Cloud Site Tubingen the allocation of the desired resources (number of virtual CPU cores, number of virtual machines (VMs), amount of storage and RAM) will be covered. Furthermore, to provide secure access to the VMs to researchers, so-called security groups will be created, which can be seen as a VM specific firewall to control incoming and outgoing network traffic connections. To provide an additional layer of security to Lux-GIANT genomics data, a private network will be added to the Lux-GIANT cloud project to protect the network traffic from and to the volume where the genomics data reside. In addition to the network traffic protection, a certificate-based approach will be used to grant specific permissions (read, write) on a per-user base. The certificates will be distributed to the particular person using a state-of-the-art asymmetric encryption technique. The whole process starting with the project application is illustrated in Figure 4. For further details, please see (37). The system-infrastructure, as created, will provide a secure environment to handle and process sensitive patient data in a restrictive and responsible way using cloud resources. To complement our cloud-based activities, the “data integration” site, ELIXIR-Luxembourg Node (ELIXIR-LU) will FAIRify this data by making them Findable, Accessible, Interoperable, and Reusable (38). All the meta-data will be shared through the ELIXIR data catalog (https://datacatalog.elixir-luxembourg.org) that facilitates the Findability of the data. Both clinical and associated molecular data will be curated, harmonized, and integrated into a discovery analytics system—Ada (https://ada-discovery.github.io). It will be hosted in the de.NBI cloud Tubingen and will facilitate the data exploration and analysis through intuitive web interface rich with dynamic visual analytics and advanced machine learning (Deep Learning).

GAP-India aims to share data at the end of a 2-year embargo period, consistent with guidelines followed by other consortia's such as H3AfricaConsortium. The purpose of the 2-year embargo period is to give Lux-GIANT researchers a reasonable time-frame to analyze and publish their data before others do. The GAP-India project aims to develop an extensive data sharing plan designed to maximize the utility of its data for the scientific community. Lux-GIANT cloud portal through which GAP-India data will share data fall into two categories: (i) controlled access, and (ii) open access.

The controlled access via the Lux-GIANT portal hosted on the de.NBI Cloud will be given to researchers/institutes who will comply with the data protection and ethical regulations, as described in the GDPR, and PDP 2019. The open-access data which does not require prior ethical clearance will be made available to the scientific community either via the Lux-GIANT Portal or PDgene database.

#### Regulatory and Ethical Framework

GAP-India project aims to address two main issues: (1) To generate the most comprehensive PD genome-phenome catalog, including iPSC biobank of the Indian PD population, and (2) to develop scientific and infrastructure capacities in India which have so far lagged in PD genomics research.

One of the major reasons that hinder the collaboration in the genomic era between various research consortia which are primarily led by institutions either in the USA and/or Europe and under-represented population such as India were concerns that data generated from the under-represented population will not be properly represented by the local stakeholders. GAP-India aims to dispel this notion of “scientific imperialism” by developing the “knowledge-sharing” model and also establishing the guidelines which adequately protect the interests of local investigators as well.

The data generated from the GAP-India project follows the strict ethical guidelines, as stipulated by the Indian Medical Council of Research (ICMR)- HMSC for international collaborative research and follows the provisions of ICMR guidelines for biomedical research in India (39). All the clinical recruiting sites obtain ethical approval from their specific ethics committee according to local protocols.

The GAP-India project involves multi-centers across India. There exists a considerable disparity in access to and protocols for regular health care among patients. Therefore, various ethical considerations have arisen during the development stage of the GAP-India project. Specifically, the following issues have been considered. (1) return of genetic results generated from the study, and how they will be received; (2) providing information about genetic findings to patients and care-providers; (3) concerns about stigmatization; and (4) ensuring equity and fairness in collaboration.

As per the Indian guidelines, we are mandated to return actionable results, with the potential to improve the health outcome of the participants. For this, a re-identification process will be followed through the PI of the recruiting center. Genetic counseling and guidance will be offered in case of such a return of results. Incidental findings that are not actionable will not be returned (39).

One of the major spin-offs from this study will create a core network of clinicians and researchers dedicated to PD genetics in India. A long-term biorepository and capacity building in terms of infrastructure and skill upgradation are additional advantages. Taken together, GAP-India aims to develop a dedicated pool of researchers and health care professionals to raise PD awareness in India.

The GAP-India study and the LUX-GIANT network aim to address a critical gap in knowledge regarding the genetic origins of PD, by leveraging the population diversity afforded by as a yet unaddressed population. We expect to generate novel data that may drive targeted therapies and make them applicable on a global scale.

## Ethics Statement

The studies involving human participants were reviewed and approved by Institutional Ethics Committees of all participating clinical centers. The patients/participants provided their written informed consent to participate in this study.

## Author Contributions

RR conceived, designed, organized and wrote the first draft, reviewed, and critically revised the manuscript. KD, RMK, and RY conceived the project, reviewed, and critically revised the manuscript. VS, UM, PA, NK, TF, HK, AS, KS, SM, SD, SK, LP, MB, PW, SR, MH, GW, and SP reviewed and critically revised the manuscript. FB, MH, JK, AK-S, SG, PL, MSt, and JR reviewed, and critically revised the manuscript. VB, GC, JS, PS, TG, OR, and VG reviewed and critically revised the manuscript. PP, RK, and RB organized the research project, and reviewed and critically revised the manuscript. KB, NC, RC, CT, MD, CG, HM, NK, SK, PM, CS, AKS, and DW reviewed and critically revised the manuscript. AK and MSh conceived, organized, and executed the research project; designed, executed, reviewed, and wrote the first draft; and reviewed and critically revised the manuscript.

## Conflict of Interest

The authors declare that the research was conducted in the absence of any commercial or financial relationships that could be construed as a potential conflict of interest.

## The Lux-Giant Collaborators

Roopa Rajan^1^, K. P. Divya^2^, Rukmini Mridula Kandadai^3^, Ravi Yadav^4^, Venkata P. Satagopam^5, 6^, U. K. Madhusoodanan^2^, Pankaj Agarwal^7^, Niraj Kumar^8^, Teresa Ferreira^9^, Hrishikesh Kumar^10^, A. V. Sreeram Prasad^11^, Kuldeep Shetty^12^, Sahil Mehta^13^, Soaham Desai^14^, Suresh Kumar^15^, L. K. Prashanth^16^, Mohit Bhatt^17^, Pettarusp Wadia^18^, Sudha Ramalingam^19^, G. M. Wali^20^, Sanjay Pandey^21^, Felix Bartusch^22^, Maximilian Hannussek^23^, Jens Krüger^22^, Ashwin Kumar-Sreelatha^23^, Sandeep Grover^23^, Peter Lichtner^24^, Marc Sturm^25^, Jochen Roeper^26^, Volker Busskamp^27^, Giriraj R. Chandak^28^, Jens Schwamborn^5^, Pankaj Seth^29^, Thomas Gasser^30^, Olaf Riess^25^, Vinay Goya^11, 31^, Pramod Kumar Pa^14^, Rupam Borgohain^3^, Rejko Krüger^5, 32^, Asha Kishore^2^, Manu Sharma^23^, Kathrin Brockmann^30, 33^, Nicolas Casadei^25^, Rituparna Chaudhari^29^, Cibin T. R.^2^, Monojit Debnath^4^, Christian Johannes Gloeckner^33^, Hardeep Malhotra^15^, Nitish Kamble^4^, Syam Krishnan^2^, Patrick May^5^, Claudia Schulte^30, 33^, Achal K. Srivastava^1^, Daniel Weiss^30, 33^.

^1^Department of Neurology, All India Institute of Medical Sciences, New Delhi, India; ^2^Sree Chitra Tirunal Institute for Medical Sciences, Trivandrum, India; ^3^Department of Neurology, Nizam's Institute of Medical Sciences, Hyderabad, India; ^4^National Institute of Mental Health and Neurosciences (NIMHANS), Bengaluru, India; ^5^Luxembourg Centre for Systems Biomedicine, University of Luxembourg, Belvaux, Luxembourg; ^6^ELIXIR-Luxembourg Node, Belvaux, Luxembourg; ^7^Movement Disorders Clinic, Global Hospitals, Mumbai, India; ^8^All India Institute of Medical Sciences, Rishikesh, India; ^9^Goa Medical College, Panaji, India; ^10^Institute of Neurosciences, Kolkata, India; ^11^Lourdes Hospital, Kochi, India; ^12^Narayana Hrudayalaya Multispeciality Hospital, Bangalore, India; ^13^Department of Neurology, PGIMER, Chandigarh, India; ^14^Shree Krishna Hospital and Pramukhswami Medical College, Karamsad, India; ^15^Department of Neurology, Vijaya Health Centre, Chennai, India; ^16^Center for Parkinson's Disease and Movement Disorders, Vikram Hospital, Bangalore, India; ^17^Kokilaben Dhirubhai Ambani Hospital, Mumbai, India; ^18^Jaslok Hospital, Mumbai, India; ^19^Department of Community Medicine, PSG Institute of Medical Sciences and Research, Coimbatore, India; ^20^Neurospecialities Centre, Belgaum, India; ^21^Department of Neurology, G. B. Pant Institute of Medical Education and Research, New Delhi, India; ^22^Zentrum für Datenverarbeitung (ZDV), University of Tubingen, Tübingen, Germany; ^23^Centre for Genetic Epidemiology, Institute for Clinical Epidemiology and Applied Biometry, University of Tubingen, Tübingen, Germany; ^24^Helmholtz Zentrum München, German Research Center for Environmental Health, Institute of Human Genetics, Neuherberg, Germany; ^25^Institute for Medical Genetics and Applied Genomics, University of Tübingen, Tübingen, Germany; ^26^Institute of Neurophysiology, Goethe University Frankfurt, Frankfurt, Germany; ^27^Department of Ophthalmology, Universitäts-Augenklinik Bonn, University of Bonn, Bonn, Germany; ^28^Centre for Cellular and Molecular Biology, Hyderabad, India; ^29^National Brain Research Centre, Manesar, India; ^30^Department of Neurodegenerative Diseases, Hertie Institute for Clinical Brain Research, University of Tübingen, Tübingen, Germany; ^31^Medanta the Medicity, Gurgaon, India; ^32^Transversal Translational Medicine, Luxembourg Institute of Health (LIH), Strassen, Luxembourg; ^33^German Center for Neurodegenerative Diseases (DZNE), Tübingen, Germany.
