# Supplementary material for: Genetic Architecture of Parkinson's Disease in the Indian Population: Harnessing Genetic Diversity to Address Critical Gaps in Parkinson's Disease Research
Source: Front Neurol. 2020 Jun 18;11:524. doi: 10.3389/fneur.2020.00524 (PMC7323575; doi:10.3389/fneur.2020.00524)
Supplement: Supplementary file 1 [file Image_1.pdf]

Supplementary Figure 1: Clinical nodal and subcenters

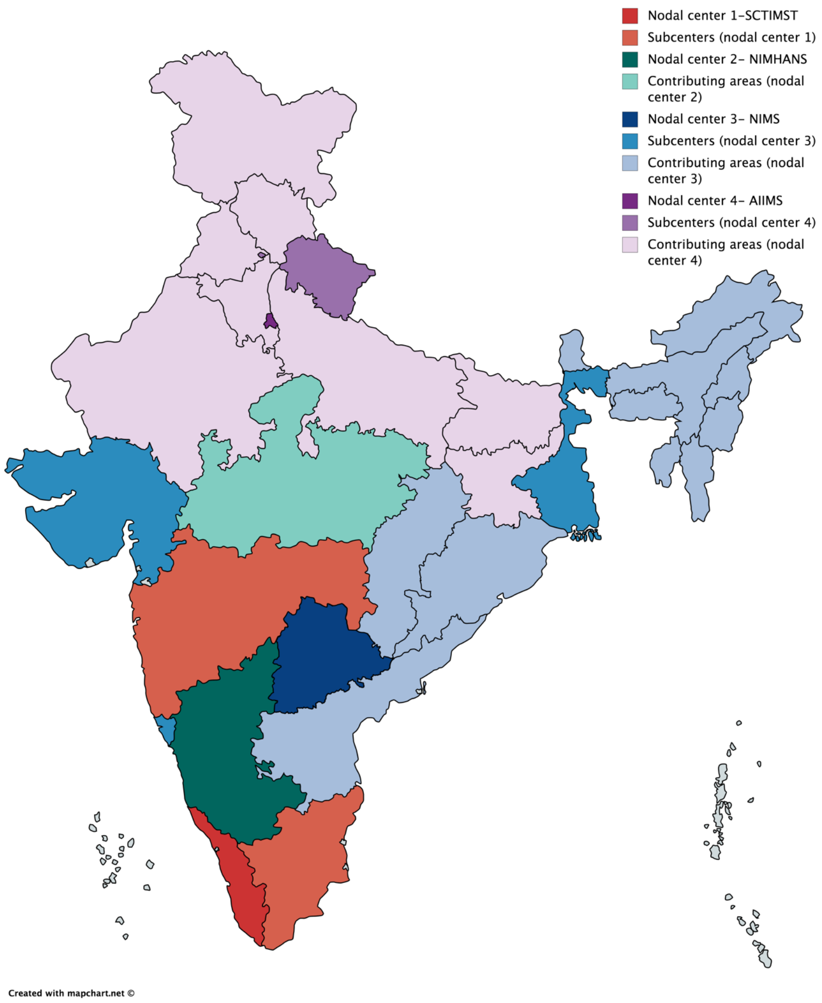

Supplementary Figure1: Geographical map of India showing distribution of nodal and subcenters for patient recruitment and bio specimen collection.

Commented [m1]: Roopa_
Please do the following to reflect our study has a broad Indian representation which is indeed the case.. Highlight nodal centre “states” with the same color. Sub-nodal centres states with different color.. Highlight states which have not been cpatured directly, but do see patients either at nodal centres or sub-noda centres… Use addiotnal color to capture those states… I think with this approach, we will map whole India… Kindly show me the figure again before submission. For example, PGI does cover Pb, Harayana, Himachal and J&K.
